# Asymmetric Strand Segregation: Epigenetic Costs of Genetic Fidelity?

**DOI:** 10.1371/journal.pgen.1000509

**Published:** 2009-06-05

**Authors:** Diane P. Genereux

**Affiliations:** Department of Biology, University of Washington, Seattle, Washington, United States of America; The Babraham Institute, United Kingdom

## Abstract

Asymmetric strand segregation has been proposed as a mechanism to minimize effective mutation rates in epithelial tissues. Under asymmetric strand segregation, the double-stranded molecule that contains the oldest DNA strand is preferentially targeted to the somatic stem cell after each round of DNA replication. This oldest DNA strand is expected to have fewer errors than younger strands because some of the errors that arise on daughter strands during their synthesis fail to be repaired. Empirical findings suggest the possibility of asymmetric strand segregation in a subset of mammalian cell lineages, indicating that it may indeed function to increase genetic fidelity. However, the implications of asymmetric strand segregation for the fidelity of epigenetic information remain unexplored. Here, I explore the impact of strand-segregation dynamics on epigenetic fidelity using a mathematical-modelling approach that draws on the known molecular mechanisms of DNA methylation and existing rate estimates from empirical methylation data. I find that, for a wide range of starting methylation densities, asymmetric—but not symmetric—strand segregation leads to systematic increases in methylation levels if parent strands are subject to de novo methylation events. I found that epigenetic fidelity can be compromised when enhanced genetic fidelity is achieved through asymmetric strand segregation. Strand segregation dynamics could thus explain the increased DNA methylation densities that are observed in structured cellular populations during aging and in disease.

## Author Summary

Through my investigations of the fidelity of epigenetic inheritance, I became intrigued by the interplay of genetic and epigenetic fidelities. Cairns proposed in 1975 that the lifetime risk of epithelial cancers would be reduced if chromosomes containing the oldest DNA strands were selectively segregated to somatic stem cells. I wondered about the implications of such asymmetric strand segregation for the fidelity of epigenetic information. To address this issue, I modelled the partitioning of DNA molecules after replication, with special attention to the molecule that contained the oldest strand. I found that the enhanced genetic fidelity that may be achieved through asymmetric strand segregation could, under some scenarios, compromise epigenetic fidelity. I am excited to pursue these studies as they apply to epigenetic changes observed to occur during aging and in human diseases, including several cancers.

## Introduction

Cairns proposed [1] that asymmetric strand segregation could help to minimize effective mutation rates in epithelial cells, which undergo frequent division and thus are highly susceptible to mutation. Under Cairns's model, after each round of DNA replication, the double-stranded molecule that contains the oldest DNA strand is preferentially targeted to the daughter cell that will be a somatic stem cell. The oldest DNA strands are expected to contain fewer errors than are daughter strands because some of the errors that arise on daughter strands during their synthesis fail to be repaired. Empirical findings suggest the possibility of asymmetric strand segregation in some [2]–[5] — but not all [6],[7] — mammalian cell lineages.

A few reports have discussed possible epigenetic causes and consequences of asymmetric strand segregation [8]–[15]. Klar [8] reported that epigenetic differences between DNA strands encode developmental asymmetries in fission yeast, and, more recently, suggested that breakdown of strand asymmetry could lead to disease in humans [10]. Merok et al. [11] noted that asymmetric strand segregation, which they report for cultured mammalian cells, could have consequences for the integrity of information encoded in epigenetic modifications of DNA. Cairns suggested that epigenetic changes to older strands could help to mark the stem cells that preferentially retain them [13], and Rando [14] proposed that epigenetic modifications, including DNA methylation, could provide information that would distinguish among DNA strands of different ages. Here, I use a population-epigenetic model of an epithelial crypt to investigate in detail the potential consequences of asymmetric strand segregation for the fidelity of epigenetic information.

## Results

**Figure 1:**
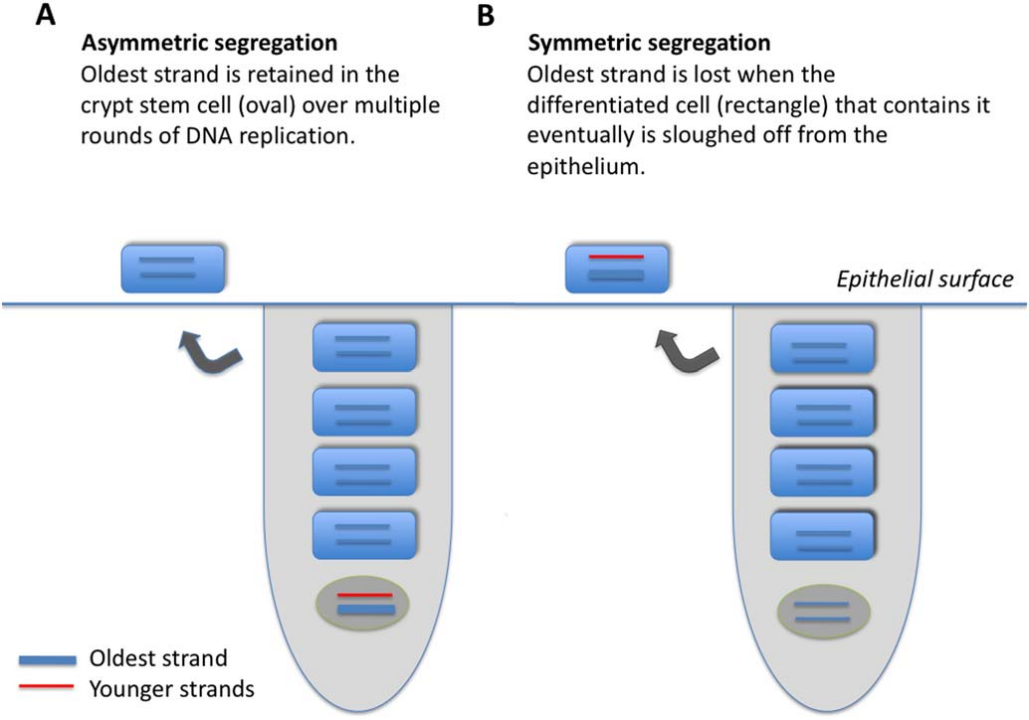
Model of two epithelial cell crypts, one showing asymmetric (A), and the other symmetric (B), strand segregation. An epithelial crypt is composed of one long-lived stem cell (oval, bottom of crypt), and four daughter cells (rectangles). The oldest DNA strand is shown as a thick line; strands produced more recently are shown as thin lines. Each round of stem-cell division, produces one terminally differentiated cell, and one stem cell; a terminally differentiated cell is sloughed off at the epithelial surface. When strand segregation is asymmetric (A), the DNA molecule containing the oldest strand is retained in the somatic stem cell over multiple rounds of DNA replication and cell division. When strand segregation is symmetric (B), the oldest strand is assigned at random to the stem cell or to the differentiated cell, and is eventually lost when the terminally differentiated cell that contains it is sloughed off at the epithelial surface.

**Figure 2:**
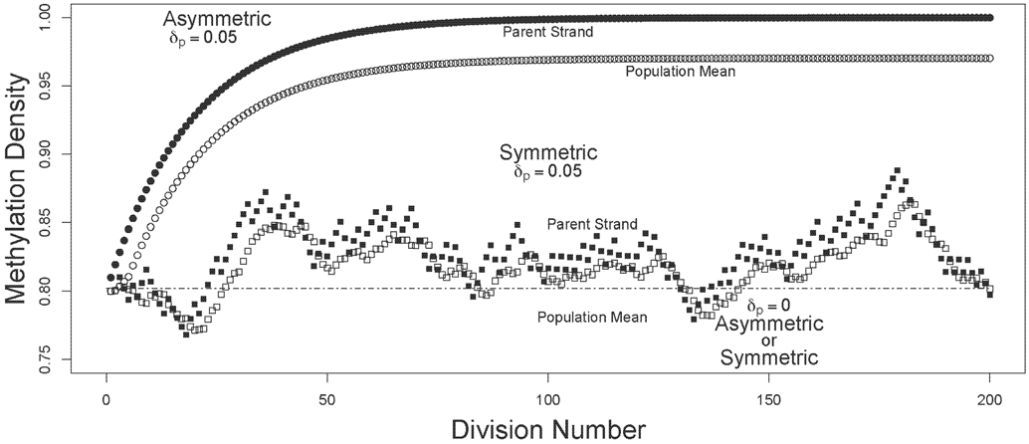
Trajectories of methylation densities under asymmetric or symmetric strand segregation, with high initial methylation density. The oldest-parent strand and the population-mean methylation densities (filled and open circles, respectively), are shown for simulations run under asymmetric and symmetric modes of strand segregation (circles and squares, respectively). For the simulations shown here, I used a starting methylation density of 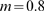. For the scenarios with parent strand de novo methylation, densities were calculated with 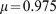, and 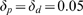. Under asymmetric strand segregation, these parameter values lead to monotonic increases in population-mean and oldest-parent strand methylation densities (upper curves). Under symmetric strand segregation, these parameter values lead to population-mean and oldest-parent strand DNA methylation densities that were dynamic about the starting value (middle curves). With no parent strand de novo methylation (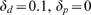), densities were unchanged under both symmetric and asymmetric strand segregation (dashed line).

**Figure 3:**
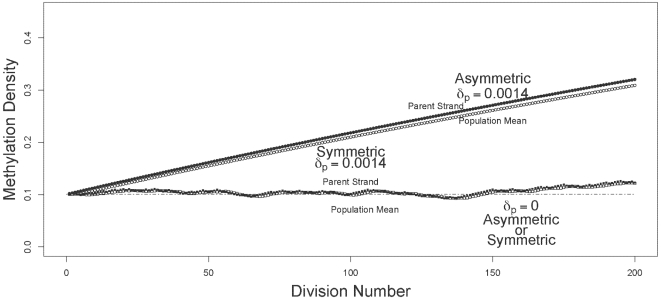
Trajectories of methylation densities under asymmetric or symmetric strand segregation, with low initial methylation density. The oldest-parent strand and the population-mean methylation densities (filled and open circles, respectively), are shown for simulations run under asymmetric and symmetric modes of strand segregation (circles and squares, respectively). The simulations shown here used a starting methylation density of 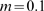. For the scenarios with parent strand de novo methylation, densities were calculated with 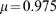, and 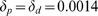. Under asymmetric strand segregation, these parameter values lead to monotonic increases in population-mean and oldest-parent strand methylation densities. Under symmetric strand segregation, these parameter values lead to population-mean and oldest-parent strand DNA methylation densities that are dynamic about the starting value. With no parent-strand de novo methylation (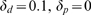), densities were unchanged under both symmetric and asymmetric strand segregation (dashed line). Note that the range and scale of the y-axis differ between Figure 2 and Figure 3.

I compared the dynamics of mean and oldest-strand methylation densities under asymmetric and symmetric strand segregation (Figure 1). Three key observations held for both high (Figure 2) and low (Figure 3) initial methylation densities: *(i)* when de novo methylation events were permitted to occur on both parent and daughter strands, asymmetric strand segregation resulted in population-mean and oldest-strand methylation densities that increased monotonically (upper curves, Figures 2 and 3); *(ii)* when de novo methylation events occured on both parent and daughter strands, symmetric segregation yielded population-mean and oldest-strand methylation densities that, although dynamic, remained very near the predicted equilibrium (middle curves, Figures 2 and 3); *(iii)* when de novo methylation events were limited to the daughter strand, population-mean and oldest-strand methylation densities under both asymmetric and symmetric segregation remained very close to starting values (dotted lines, Figures 2 and 3). Thus, for a wide range of starting methylation densities, asymmetric — but not symmetric — strand segregation leads to systematic increases in methylation levels, if parent strands are subject to de novo methylation events.

## Discussion

The population-epigenetic model I develop here reveals that asymmetric strand segregation in somatic stem cells could lead to monotonic increases in DNA methylation densities in structured cellular populations. These increases are predicted to occur when de novo methylation occurs on parent as well as daughter strands, but not when de novo methylation events are limited to the daughter strand.

The predictions of my model are made using empirical estimates of methylation rates in differentiated cells, for which substantial amounts of data are available. Further work will be necessary directly to ascertain methylation rates in somatic stem cells. Nevertheless, the essential findings of my study are consistent across a broad range of parameter values (see, for instance, Figures 2 and 3), suggesting that these results will hold even if methylation densities and rates differ appreciably between differentiated and somatic stem cells.

The accumulation of aberrant methylation predicted by my model may have different time courses depending on the biological properties of a given lineage of somatic stem cells, and on the initial methylation density of a given locus. When somatic stem cell division always gives rise to one stem cell and one differentiated cell, as I model here, substantial increases in DNA methylation densities can occur over just a few cell divisions (Figures 2). When somatic stem cell division sometimes gives rise to one stem cell and one differentiated cell, and sometimes to two somatic stem cells, somewhat lower rates of increase could occur. The rate of increase will also depend on the initial DNA methylation density (compare, for instance, Figures 2 and 3). Lorincz et al. [16] found that progression to dense methylation is especially likely for genomic regions that have already attained intermediate methylation densities. In light of this finding, it seems plausible that even slow or transient increases in DNA methylation could raise methylation densities to a threshold sufficient to trigger more substantial increases.

What might be the functional implications of the increased DNA methylation densities predicted under asymmetric strand segregation? The accumulation of methyl groups on a long-lived DNA strand could serve as a signal to guide asymmetric strand segregation itself [17], or to distinguish stem cells from differentiated cells [13]. My findings could also help to explain the positive correlation observed between age and methylation density in endometrial [18] and intestinal [19] tissues. Both of these are rapidly-dividing tissues of the sort initially predicted by Cairns [1], and reported by some groups [4], to undergo asymmetric strand segregation. In contrast, slowly-dividing cells, such as those in the hematopoetic lineage, have constant methylation densities [20]–[23] and have been reported not to undergo asymmetric strand segregation [6]. Thus, the systematic increases in DNA methylation densities predicted here may be specific to the rapidly-dividing lineages Cairns initially discussed [1].

My results may also have implications for the etiology of cancer in humans. Several epithelial cancers are associated with reductions in epigenetic fidelity, including the accumulation of aberrant methylation and abnormal gene silencing [24],[25]. Barrett's esophagus illustrates the potential relevance of these findings. The esophageal epithelium in Barrett's esophagus contains abnormal intestinal crypt-like structures, and is characterized by abrupt increases in DNA methylation densities and consequent silencing of loci critical to cell-cycle regulation [26].

Thus, it is possible that directional change in epigenetic information may be a cost of the increased genetic fidelity achieved through asymmetric strand segregation, with implications for human disease.

## Models

### Modelling an Epithelial Crypt

I developed a simplified model of an epithelial crypt with which to track methylation dynamics (Figure 1). Each crypt consists of one somatic stem cell, and four differentiated cells. At each round of stem-cell division, one terminally differentiated cell is produced, and one stem cell is produced. The top-most of the terminally differentiated cells is sloughed off at the epithelial surface. Segregation of the oldest DNA strand always to the stem cell characterizes asymmetric strand segregation (Figure 1a); segregation of the oldest DNA strand at random to the stem and terminally differentiated cells characterizes symmetric segregation (Figure 1B).

### Modelling DNA Methylation Events in an Epithelial Crypt

#### Maintenance and de novo methylation

I modelled replication and methylation dynamics for a single, methylated locus such as one of those on the hemizygous X chromosome in a human male, or on the inactive X chromosome in a human female. Prior to cell division, the locus undergoes semi-conservative replication, producing two double-stranded DNA molecules. Each molecule is composed of a parent strand from the original double-stranded molecule, and a newly-synthesized daughter strand. The model to be described in detail below compares methylation dynamics under asymmetric (Figure 1A) and symmetric (Figure 1B) strand segregation.

Methyl groups are added to CpG cytosines in DNA by two different processes: maintenance methylation and de novo methylation. Maintenance methylation is performed by maintenance methyltranferases, which exhibit a preference for hemimethylated CpG/CpG dyads, are thought to localize to the replication fork, and operate principally during DNA replication [27]. De novo methyltransferases do not seem to exhibit this preference for hemimethylated sites, and may be active throughout the mammalian cell cycle [28]–[30].

Here, the maintenance methylation rate, 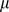, is defined as the probability that a methyl group is added to a daughter-strand CpG, given that the complementary CpG on the parent strand is methylated. Maintenance events produce fully methylated CpG/CpG dyads. Maintenance methylation fails at rate 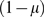, yielding hemimethylated dyads that have a methylated CpG on the parent strand, and an unmethylated, complementary CpG on the daughter strand [31]. The daughter strand de novo methylation rate, 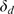, is defined as the probability that a methyl group is added to a daughter-strand CpG that remains unmethylated after DNA replication and maintenance methylation. The parent strand de novo methylation rate, 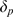, is defined as the probability that a methyl group is added to an unmethylated CpG on the parent strand. I assume, as before [31], that methyl groups are not actively removed from DNA in differentiated cells, consistent with the lack of evidence that active demethylation occurs in epithelial cell lineages. My results would not apply in cases where there is net loss of DNA methylation from individual DNA strands, such as in early mammalian development [32].

The question of whether or not de novo methylation events can occur *in vivo* on the parent strand was addressed experimentally during the 1970s, yielding conflicting data [33]–[36]. More recent modelling and statistical studies of epigenetic fidelity have used a variety of approaches to accommodate this continued uncertainty. Otto and Walbot [37] assumed that de novo methylation occurs simultaneously on parent and daughter strands. In our previous model [31], we considered two scenarios: that de novo methylation occurs independently and at equal rates on the two strands, and alternately, that it is limited to the daughter strand. A new statistical study by Fu et al. (Fu et al., Manuscript in Preparation) found far greater support for the presence of parent-strand de novo methylation than for its absence, at least in lymphocytes from adult humans. Because it remains unclear whether de novo methylation on the parent strand is a universal phenomenon or whether it is, instead, limited to a subset of cell lineages or developmental stages, I here consider methylation dynamics both with and without parent-strand de novo methylation.

### Mathematical Model of DNA Methylation Events and Strand Segregation in an Epithelial Crypt



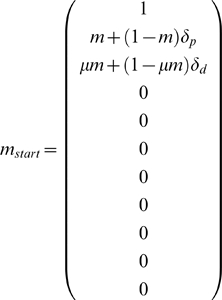
(1)


I developed vector 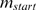 (1) to record the methylation density of each of the 10 individual strands of the five DNA molecules in the epithelial crypts (Figure 1). The initial state of the epithelial crypt is given by 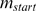.

The first element of 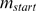 (1), “1”, is used to implement the assumption that individual methyl groups are not lost once they are incorporated. Elements two through eleven represent the methylation densities of the ten individual strands of DNA in the five double-stranded molecules of the crypt cells. These elements are best considered in pairs. Elements two and three represent the methylation states of the parent and daughter strands in the founding somatic stem cell. In particular, the second element gives the methylation density of the oldest parent strand at the start of the simulation. This strand is assumed to have started with methylation density 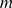, as calculated under our previous model [31] from the chosen parameter values for maintenance and de novo methylation events. It then acquired additional methyl groups through parent strand de novo methylation events occurring at rate 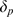, for those cases where 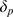 is greater than 0. The third element gives the initial methylation density of the daughter strand in the founding somatic stem cell, as calculated from the starting methylation density of the parent strand, 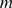, using our earlier model [31]. Elements four and five represent methylation states of the parent and daughter strands in the first of the four differentiated cells, and so on up to elements ten and eleven, which give the methylation states of the parent and daughter strands in the differentiated cell closest to the epithelial surface. Because I start by modelling the establishment of the crypt, the strands represented by vector positions three through eleven have not yet been synthesized at the start of the simulation, and therefore have methylation densities of zero. Methylation densities of strands that do not yet exist are excluded from the calculation of population-mean densities.
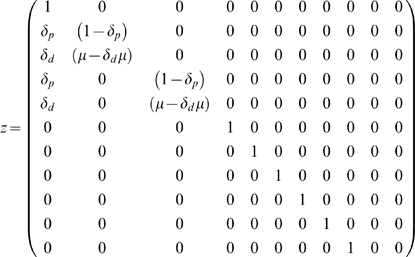
(2)


I developed matrix 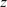 (2) to model the occurrence of methylation events on individual strands of DNA molecules, and to track their progression through the simplified epithelial crypt described above.

The first row of matrix 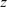 is a placeholder, and is used to regenerate the “1” that is the first element of vector 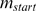. The second row of the matrix is used to calculate the updated methylation density of the oldest DNA strand. The third row of the matrix is used to simulate methylation events on the daughter strand that has just been produced through replication of the oldest DNA strand. This density is determined by the maintenance methylation rate, 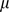, and the daughter-strand de novo methylation rate, 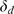. The fourth row of the matrix is used to simulate methylation events on the parent strand in the newest differentiated cell, and records methylation events that occur through parent-strand de novo methylation, at rate 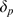. The fifth row of the matrix is used to simulate methylation events on the daughter strand in the newest differentiated cell, and records methylation events that occur by daughter-strand de novo methylation, at rate 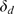. The sixth through eleventh rows of the matrix are used to simulate the progression of existing DNA molecules through the crypt. As noted above, we assume that methyl groups are never removed from a strand once they have been added, and are added to a strand either during the round in which it is synthesized, or during subsequent rounds in which it serves as a parent strand in DNA replication. Rows 6, 8 and 10 simulate the movements of strands that are parents in their respective cells; rows 7, 9 and 11 simulate the movements of strands that are daughters in their respective cells. Upon replication and cell division, cells containing the various DNA molecules advance one cell-position toward the epithelial surface.

Both asymmetric and symmetric strand segregation are modelled using matrix 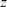, with slight differences in the treatment of the resulting updated 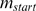. To model a single round of DNA replication and cell division under *asymmetric* strand segregation, I multiplied matrix 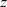 by vector 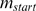. To investigate methylation trajectories over multiple rounds of DNA replication and cell division, I multiplied matrix 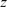 by vector 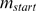 recursively up to 500 times. This large number of divisions would be unreasonable for many tissues, but is likely appropriate for the rapidly-dividing cells of the endometrium [18] and intestinal epithelium [38] over a period of several years [39].

To model a single round of DNA replication and cell division under *symmetric* strand segregation, I used a similar approach, multiplying matrix 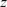 by vector 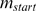, but included for each round a random draw of a number between 0 and 1. When the random number was less than or equal to 0.5, I retained the vector that resulted from this initial multiplication, simulating retention of the oldest DNA strand in the somatic stem cell. When the random number was greater than 0.5, I simulated the export of the oldest strand to the differentiated daughter cell by exchanging the methylation densities given in vector positions two and three with those given in vector positions four and five. I repeated this process of multiplication, random number selection, and vector rearrangement for up to 500 rounds of DNA replication, methylation events, and cell division.

### Choice of Parameter Values for Maintenance and De Novo Methylation Events

Several authors have investigated the rate of maintenance methylation, 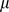, yielding estimates that range from 0.95 to 0.999 [31], [40]–[43] (Fu et al., Manuscript in Preparation). Here, I assumed 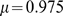.

Comparatively few studies have investigated the rate of de novo methylation. The estimates that do exist exhibit substantial variation. Pfeifer et al. [40] estimated a de novo methylation rate of 0.05, Laird et al. [41] estimated a rate of 0.17, under the assumption that de novo events are limited to the daughter strand. Results from Genereux et al. [31] suggest that part of the variation in these estimates of the de novo methylation rate may be attributable to *bona fide* biological variation among CpG cytosine sites, and perhaps among loci.

For de novo methylation rates, I first chose parameter values that yield an expected equilibrium methylation density of 0.8 under the model described previously [31], and assuming 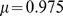. To meet this condition, the sum of parent, 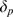 and daughter, 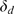, de novo rates must be 0.1. To accommodate uncertainties about the strandedness of de novo methylation events, I explored two cases: de novo events occurring on the daughter strand only (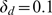, 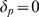), and de novo events occurring at equal rates on the parent and daughter strands (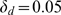, 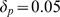). These values were used to generate the results in Figure 2. To investigate whether or not the change in methylation density observed when starting with 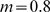 was limited to scenarios with high initial densities, I also conducted simulations spanning a range of initial values. For the simulation shown in Figure 3, I started with a methylation density of 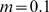, and assumed the maintenance rate, 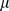, to be 0.975. To meet these conditions under our previous model [31], the sum of parent, 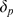, and daughter, 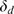, de novo rates must be 0.0028. Here, as before, I considered parameter values that included de novo methylation events on the parent strand (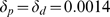), and parameter values that limited de novo methylation events to the daughter strand (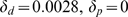).

## References

[1] Cairns J (1975). Mutation, selection and the natural history of cancer.. Nature.

[2] Lark KG, Consigli RA, Minocha HC (1966). Segregation of sister chromatids in mammalian cells.. Science.

[3] Karpowicz P, Morshead C, Kam A, Jervis E, Ramunas J (2005). Support for the immortal strand hypothesis: neural stem cells partition DNA asymmetrically *in vitro*.. J Cell Biol.

[4] Smith GH (2005). Label-retaining epithelial cells in mouse mammary gland divide asymmetrically and retain their template DNA strands.. Development.

[5] Shinin V, Gayraud-Morel B, Gomes D, Tajbakhsh S (2006). Asymmetric division and cosegregation of template DNA strands in adult muscle satellite cells.. Nat Cell Biol.

[6] Kiel MJ, He S, Ashkenazi R, Gentry SN, Teta M (2007). Haematopoietic stem cells do not asymmetrically segregate chromosomes or retain BrdU.. Nature.

[7] Sotiropoulou P, Candi A, Blanpain C (2008). The majority of multipotent epidermal stem cells do not protect their genome by asymmetrical chromosome segregation.. Stem Cells.

[8] Klar AJ (1987). Differentiated parental DNA strands confer developmental asymmetry on daughter cells in fission yeast.. Nature.

[9] Klar AJ (1994). A model for specification of the left-right axis in vertebrates.. Trends Genet.

[10] Klar AJS (2004). A genetic mechanism implicates chromosome 11 in schizophrenia and bipolar diseases.. Genetics.

[11] Merok JR, Lansita JA, Tunstead JR, Sherley JL (2002). Cosegregation of chromosomes containing immortal DNA strands in cells that cycle with asymmetric stem cell kinetics.. Cancer Res.

[12] Liu SV (2005). Linking DNA aging with cell aging and combining genetics with epigenetics.. Logical Biology.

[13] Cairns J (2006). Cancer and the immortal strand hypothesis.. Genetics.

[14] Rando TA (2007). The immortal strand hypothesis: segregation and reconstruction.. Cell.

[15] Lew DJ, Burke DJ, Dutta A (2008). The immortal strand hypothesis: how could it work?. Cell.

[16] Lorincz MC, Schubeler D, Hutchinson SR, Dickerson DR, Groudine M (2002). DNA methylation density inuences the stability of an epigenetic imprint and Dnmt3a/b-independent de novo methylation.. Mol Cell Biol.

[17] Lansdorp PM (2007). Immortal strands? Give me a break.. Cell.

[18] Kim JY, Tavare S, Shibata D (2005). Counting human somatic cell replications: methylation mirrors endometrial stem cell divisions.. Proc Natl Acad Sci U S A.

[19] Kim JY, Beart RW, Shibata D (2005). Stability of colon stem cell methylation after neo-adjuvant therapy in a patient with attenuated familial adenomatous polyposis.. BMC Gastroenterol.

[20] Stöger R, Kajimura TM, Brown WT, Laird CD (1997). Epigenetic variation illustrated by DNA methylation patterns of the fragile-X gene FMR1.. Hum Mol Genet.

[21] Bjornsson HT, Sigurdsson MI, Fallin MD, Irizarry RA, Aspelund T (2008). Intra-individual change over time in DNA methylation with familial clustering.. JAMA.

[22] Sandovici I, Naumova AK, Leppert M, Linares Y, Sapienza C (2004). A longitudinal study of X-inactivation ratio in human females.. Hum Genet.

[23] Sandovici I, Leppert M, Hawk PR, Suarez A, Linares Y (2003). Familial aggregation of abnormal methylation of parental alleles at the *IGF2/H19* and *IGF2R* differentially methylated regions.. Hum Mol Genet.

[24] Wong DJ, Foster SA, Galloway DA, Reid BJ (1999). Progressive region-specific de novo methylation of the *p16* CpG island in primary human mammary epithelial cell strains during escape from M(0) growth arrest.. Mol Cell Biol.

[25] Grady WM (2005). Epigenetic events in the colorectum and in colon cancer.. Biochem Soc Trans.

[26] Smith E, De Young NJ, Pavey SJ, Hayward NK, Nancarrow DJ (2008). Similarity of aberrant DNA methylation in Barrett's esophagus and esophageal adenocarcinoma.. Mol Cancer.

[27] Leonhardt H, Page AW, Weier HU, Bestor TH (1992). A targeting sequence directs DNA methyltransferase to sites of DNA replication in mammalian nuclei.. Cell.

[28] Okano M, Bell DW, Haber DA, Li E (1999). DNA methyltransferases Dnmt3a and Dnmt3b are essential for de novo methylation and mammalian development.. Cell.

[29] Yokochi T, Robertson KD (2002). Preferential methylation of unmethylated DNA by mammalian de novo DNA methyltransferase Dnmt3a.. J Biol Chem.

[30] Hsieh CL (1999). *In vivo* activity of murine de novo methyltransferases, Dnmt3a and Dnmt3b.. Mol Cell Biol.

[31] Genereux DP, Miner BE, Bergstrom CT, Laird CD (2005). A population-epigenetic model to infer site-specific methylation rates from double-stranded DNA methylation patterns.. Proc Natl Acad Sci U S A.

[32] Razin A, Webb C, Szyf M, Yisraeli J, Rosenthal A (1984). Variations in DNA methylation during mouse cell differentiation *in vivo* and *in vitro*.. Proc Natl Acad Sci U S A.

[33] Adams RL (1971). Methylation of newly synthesized and older deoxyribonucleic acid.. Biochem J.

[34] Schneiderman MH, Billen D (1973). Methylation rapidly reannealing DNA during the cell cycle of Chinese hamster cells.. Biochim Biophys Acta.

[35] Bird AP (1978). Use of restriction enzymes to study eukaryotic DNA methylation: II. the symmetry of methylated sites supports semi-conservative copying of the methylation pattern.. J Mol Biol.

[36] Kappler JW (1970). The kinetics of DNA methylation in cultures of a mouse adrenal cell line.. J Cell Physiol.

[37] Otto SP, Walbot V (1990). DNA methylation in eukaryotes: kinetics of demethylation and de novo methylation during the life cycle.. Genetics.

[38] Nicolas P, Kim KM, Shibata D, Tavare S (2007). The stem cell population of the human colon crypt: analysis *via* methylation patterns.. PLoS Comput Biol.

[39] Potten CS (1998). Stem cells in gastrointestinal epithelium: numbers, characteristics and death.. Philos Trans R Soc Lond B Biol Sci.

[40] Pfeifer GP, Steigerwald SD, Hansen RS, Gartler SM, Riggs AD (1990). Polymerase chain reactionaided genomic sequencing of an X chromosome-linked CpG island: methylation patterns suggest clonal inheritance, CpG site autonomy, and an explanation of activity state stability.. Proc Natl Acad Sci U S A.

[41] Laird CD, Pleasant ND, Clark AD, Sneeden JL, Hassan KMA (2004). Hairpin-bisulfite PCR: assessing epigenetic methylation patterns on complementary strands of individual DNA molecules.. Proc Natl Acad Sci U S A.

[42] Hermann A, Goyal R, Jeltsch A (2004). The Dnmt1 DNA-(cytosine-C5)-methyltransferase methylates DNA processively with high preference for hemimethylated target sites.. J Biol Chem.

[43] Vilkaitis G, Suetake I, Klimasauskas S, Tajima S (2005). Processive methylation of hemimethylated CpG sites by mouse Dnmt1 DNA methyltransferase.. J Biol Chem.

